# Supplementary material for: Active pathways of anaerobic methane oxidization in deep-sea cold seeps of the South China Sea
Source: Microbiol Spectr. 2023 Nov 2;11(6):e02505-23. doi: 10.1128/spectrum.02505-23 (PMC10715046; doi:10.1128/spectrum.02505-23)
Supplement: Supplemental material — Tables S1 to S3; Fig. S1 to S4. [file spectrum.02505-23-s0001.docx]

Supplementary materials

Active pathways of anaerobic methane oxidization in deep-sea cold seeps of the South China Sea

Qiuyun Jiang^a,e^, Hongmei Jing^a,c,d*^, Xuegong Li^a^, Ye Wan^a^, I-Ming Chou^a^, Lijun Hou^b^, Hongpo Dong^b^, Yuhui Niu^b^, Dengzhou Gao^b^

^a^ CAS Key Laboratory for Experimental Study under Deep-sea Extreme Conditions, Institute of Deep-sea Science and Engineering, Chinese Academy of Sciences, Sanya, 572000, China;

^b^ State Key Laboratory of Estuarine and Costal Research, East China Normal University, 500 Dongchuan Road, Shanghai 200241, China;

^c^ Southern Marine Science and Engineering Guangdong Laboratory (Zhuhai), 519000, China;

^d^ HKUST-CAS Sanya Joint Laboratory of Marine Science Research, Chinese Academy of Sciences, Sanya, 572000, China

^e^ University of Chinese Academy of Sciences, Beijing, 100049, China

**Corresponding Author**

* Hongmei Jing

Email: [hmjing@idsse.ac.cn](mailto:hmjing@idsse.ac.cn)

**Contents of this file**

Supplementary materials include 8 pages, 3 tables and 4 figures.

**Table S1** Primers for PCR and qPCR used in this study.

| Primers | Targeted Microbial Genes | Methods | Sequence (5′-3′) | References |
| --- | --- | --- | --- | --- |
| A189_b F | NC10 *pmo*A | PCR | GGN GAC TGG GAC TTY TGG | (Luesken et al., 2011; Ettwig et al., 2009) |
| cmo682R |  | PCR | AAA YCC GGC RAA GAA CGA |  |
| cmo182F |  | PCR | TCA CGT TGA CGC CGA TCC |  |
| cmo568R |  | PCR | GCA CAT ACT CCA TCC CCA TC |  |
| qP1F |  | qPCR | GGG CTT GAC ATC CCA CGA ACC TG |  |
| qP2R |  | qPCR | CTC AGC GAC TTC GAG TAC AG |  |
| McrA169F | ANME-2d *mcr*A | PCR | GCA GCA ATC ACC AAG AAG AGA GG | (Vaksmaa et al., 2017) |
| McrA997F |  | PCR | ATC TGG CTC GGY GGC TAC ATG T |  |
| McrA1360R |  | PCR | TGC CTC TTT GTG GAG GTA CAT GGA |  |
| AAA641F |  | qPCR | ACT GDT AGG CTT GGG ACC |  |
| AAA834R |  | qPCR | ATG CGG TCG CAC CGC ACC TG |  |
| DSR1F | *dsr*B | PCR | ACS CAC TGG AAG CAC G | (Rampinelli et al., 2008;  Girguis et al., 2005) |
| DSRp2060F |  | PCR | CAA CAT CGT YCA YAC CCA GGG |  |
| DSR4R |  | PCR | GTG TAG CAG TTA CCG CA |  |
| DSRB-213f |  | qPCR | GTT GTT TGG AGA TGA GCC C |  |
| DSRB-658r |  | qPCR | ATT CCA CTT CCT TCT CCC ATA |  |
| Bac16S_F | 16S rRNA | PCR, qPCR | ACT CCT ACG GGA GGC AGC AG | (Liu et al., 2016) |
| Bac16S_R |  | PCR, qPCR | GGA CTA CHV GGG TWT CTA AT |  |
| Arc16S_F |  | PCR, qPCR | CCC TAY GGG GYG CAS CAG | (Takai and Horikoshi, 2000) |
| Arc16S_R |  | PCR, qPCR | GGA CTA CVS GGG TAT CTA AT |  |

**Table S2** Sequences numbers of ASVs associated with different functional groups in the prokaryotic communities. I: initial; C: ^13^CH_4_; N2: ^13^CH_4_ + NO_2_^-^; N3: ^13^CH_4_ + NO_3_^-^; S: ^13^CH_4_ + SO_4_^2-^.

| Taxon | Site F | | | | | Haima | | | | | Xisha | | | | |
| --- | --- | --- | --- | --- | --- | --- | --- | --- | --- | --- | --- | --- | --- | --- | --- |
|  | I | N2 | N3 | S | C | I | N2 | N3 | S | C | I | N2 | N3 | S | C |
| Desulfobacteraceae | 290 | 6 | 36 | 16 | 19 | 0 | 0 | 0 | 0 | 0 | 0 | 0 | 0 | 0 | 0 |
| Desulfosarcinaceae | 1031 | 135 | 267 | 233 | 150 | 347 | 61 | 65 | 63 | 53 | 176 | 53 | 28 | 6 | 78 |
| Desulfobulbus | 536 | 26 | 144 | 233 | 199 | 55 | 14 | 8 | 0 | 0 | 0 | 0 | 0 | 0 | 0 |
| Desulfococcus | 0 | 0 | 0 | 0 | 0 | 3 | 0 | 0 | 0 | 0 | 0 | 0 | 0 | 0 | 0 |
| Methylomirabilaceae(NC10) | 0 | 0 | 20 | 0 | 0 | 0 | 0 | 2 | 3 | 23 | 7271 | 577 | 4547 | 1695 | 1918 |
| ANME-1a | 19 | 0 | 0 | 0 | 23 | 42 | 6 | 0 | 0 | 40 | 0 | 0 | 0 | 0 | 0 |
| ANME-1b | 0 | 10 | 15 | 0 | 14 | 424 | 136 | 8 | 37 | 247 | 0 | 0 | 0 | 0 | 0 |
| ANME-2a-2b | 13076 | 16714 | 6911 | 13997 | 5855 | 5759 | 450 | 142 | 284 | 789 | 0 | 41 | 0 | 0 | 6 |
| ANME-2c | 3050 | 4471 | 4367 | 1913 | 2051 | 1520 | 739 | 9 | 355 | 771 | 31 | 15 | 20 | 28 | 4 |
| ANME-2d | 0 | 0 | 0 | 0 | 0 | 13 | 0 | 0 | 5 | 0 | 0 | 0 | 0 | 0 | 0 |
| ANME-3 | 14032 | 18550 | 10281 | 10578 | 29971 | 7797 | 1421 | 77 | 1256 | 3444 | 0 | 90 | 22 | 22 | 0 |

**Table S3** Pearson Correlation Matrix among the concentration of nitrites, nitrates and sulfates in culture supernatant and CO_2_ production during 14-day incubation. Bold values in the table represent the better positive correlation between the variables (*p* < 0.05).

| Variables | NO_2_^-^ | CO_2_ (NO_2_^-^) | NO_3_^-^ | CO_2_ (NO_3_^-^) | SO_4_^2-^ | CO_2_ (SO_4_^2-^) |
| --- | --- | --- | --- | --- | --- | --- |
| NO_2_^-^ | 1.000 |  |  |  |  |  |
| CO_2_ (NO_2_^-^) | -0.323 | 1.000 |  |  |  |  |
| NO_3_^-^ | 0.467 | **-0.667** | 1.000 |  |  |  |
| CO_2_ (NO_3_^-^) | -0.381 | **0.893** | **-0.682** | 1.000 |  |  |
| SO_4_^2-^ | -0.483 | -0.058 | 0.301 | -0.213 | 1.000 |  |
| CO_2_ (SO_4_^2-^) | -0.289 | **0.987** | -0.612 | **0.915** | -0.095 | 1.000 |


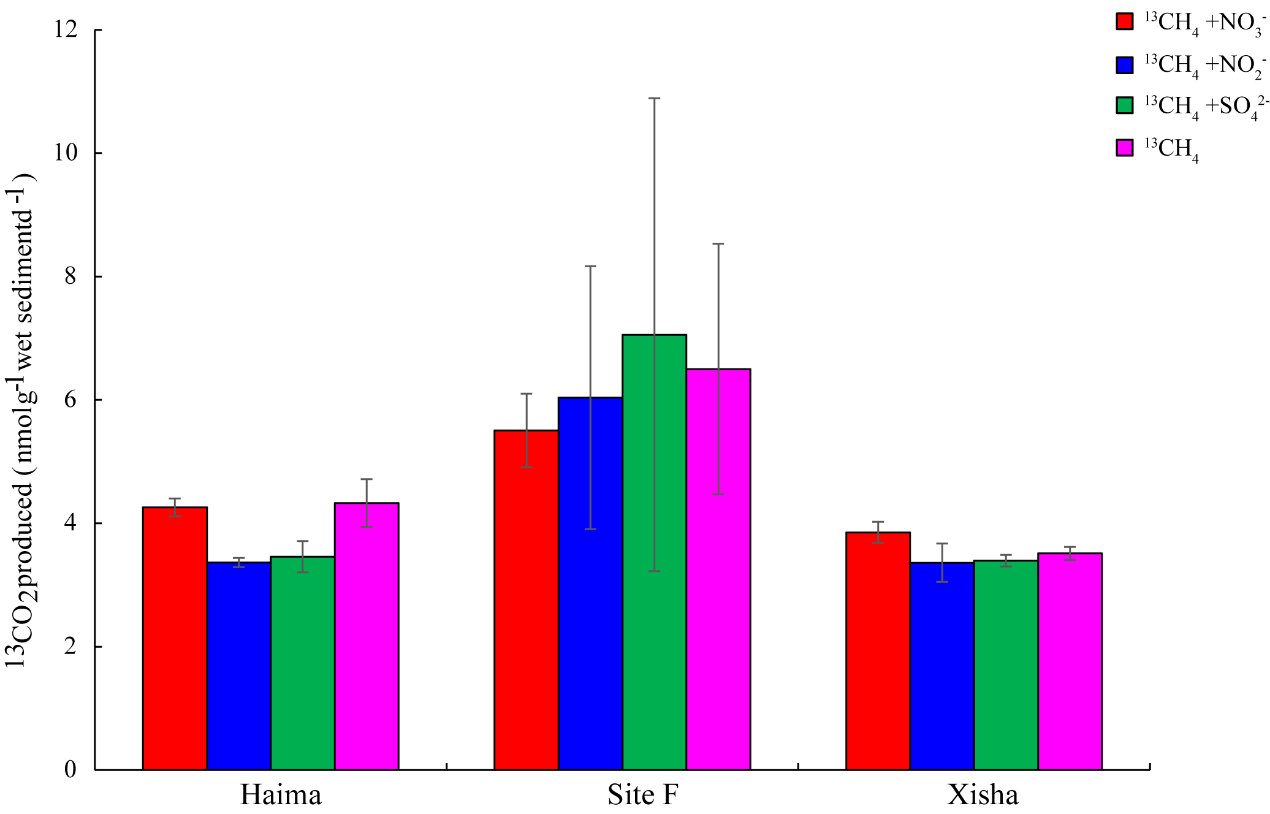


**Figure S1.** Accumulation of ^13^CO_2_ production in cold seeps and trough after14-day incubation. The values of three stations in the same region were averaged.


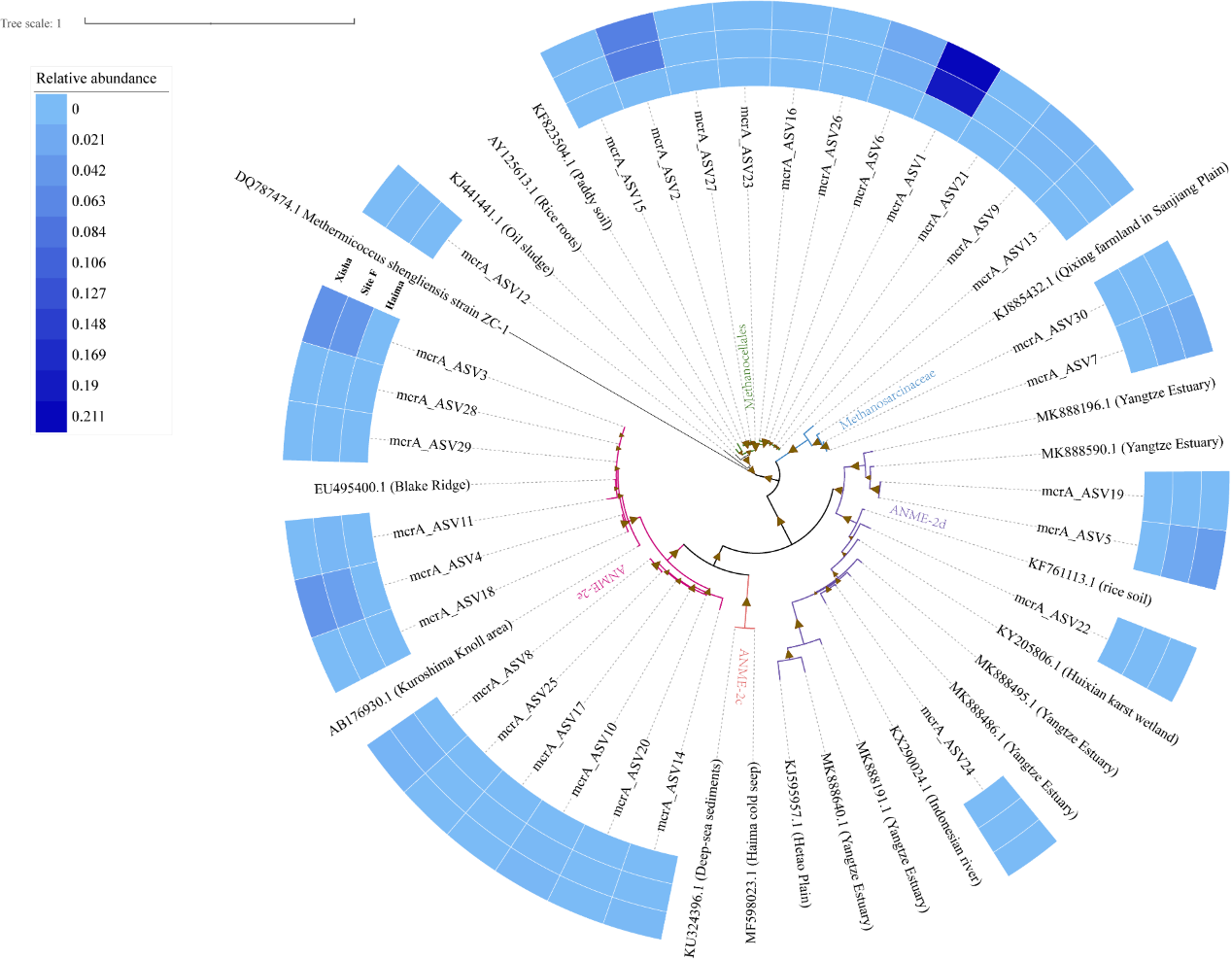


**Figure S2.** Maximum-likelihood phylogenetic trees for *mcr*A gene with top 30 ASVs (accounting for > 96.8% of the total retrieved *mcr*A gene sequences) in ^13^CH_4_ + NO_3_^-^ treatment groups (A). Bootstrap values over 50% based on 1,000 replicates were shown. The abundance of the ASVs in each region was shown in the heatmap referred to the color key.


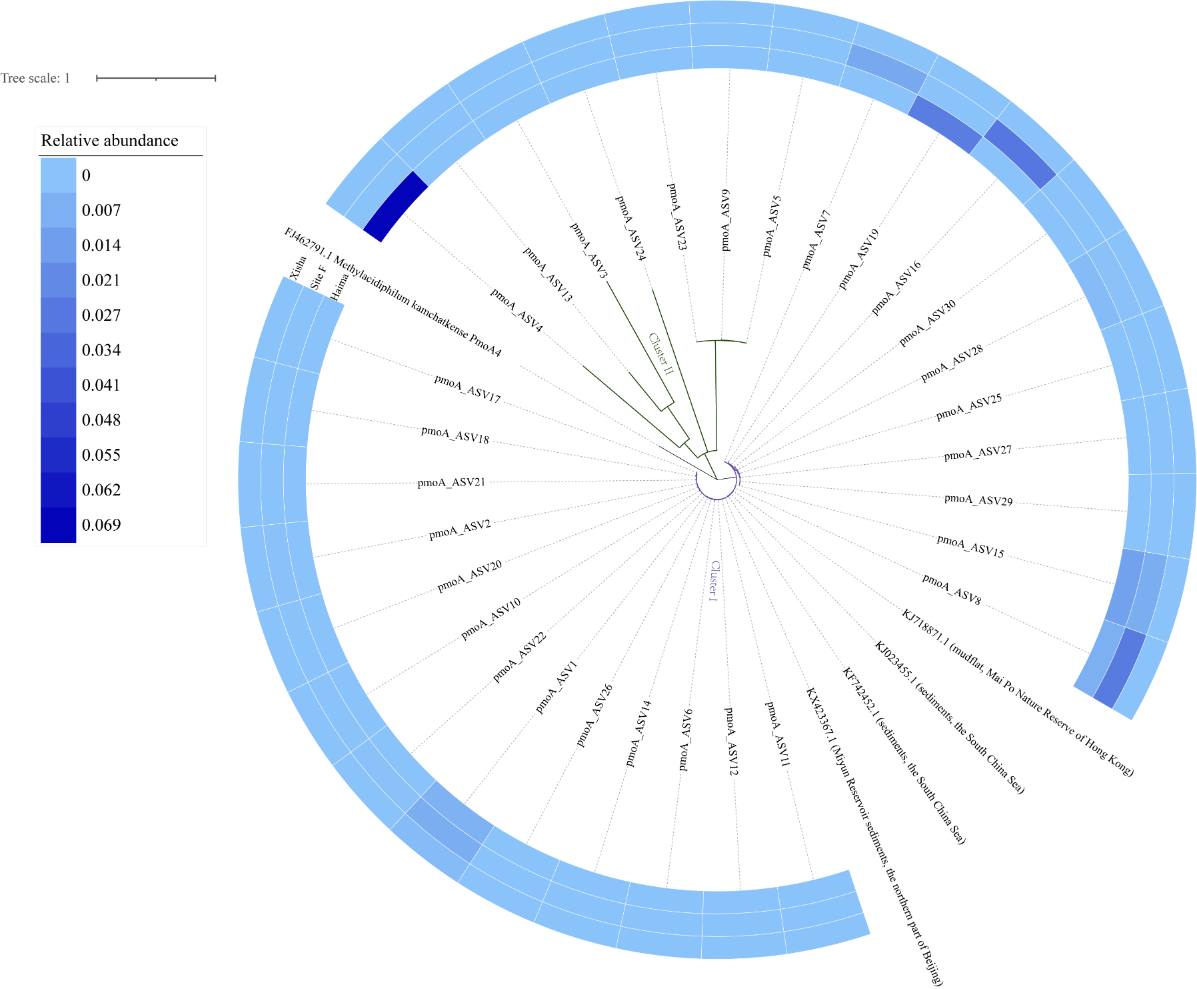


**Figure S3.** Maximum-likelihood phylogenetic trees for *pmo*A gene of top 30 ASVs (accounting for > 73.6% of the total retrieved *pmo*A gene sequences) in ^13^CH_4_ + NO_2_^-^ treatment groups. Bootstrap values over 50% based on 1,000 replicates were shown. The abundance of the ASVs in each region was shown in the heatmap referred to the color key.


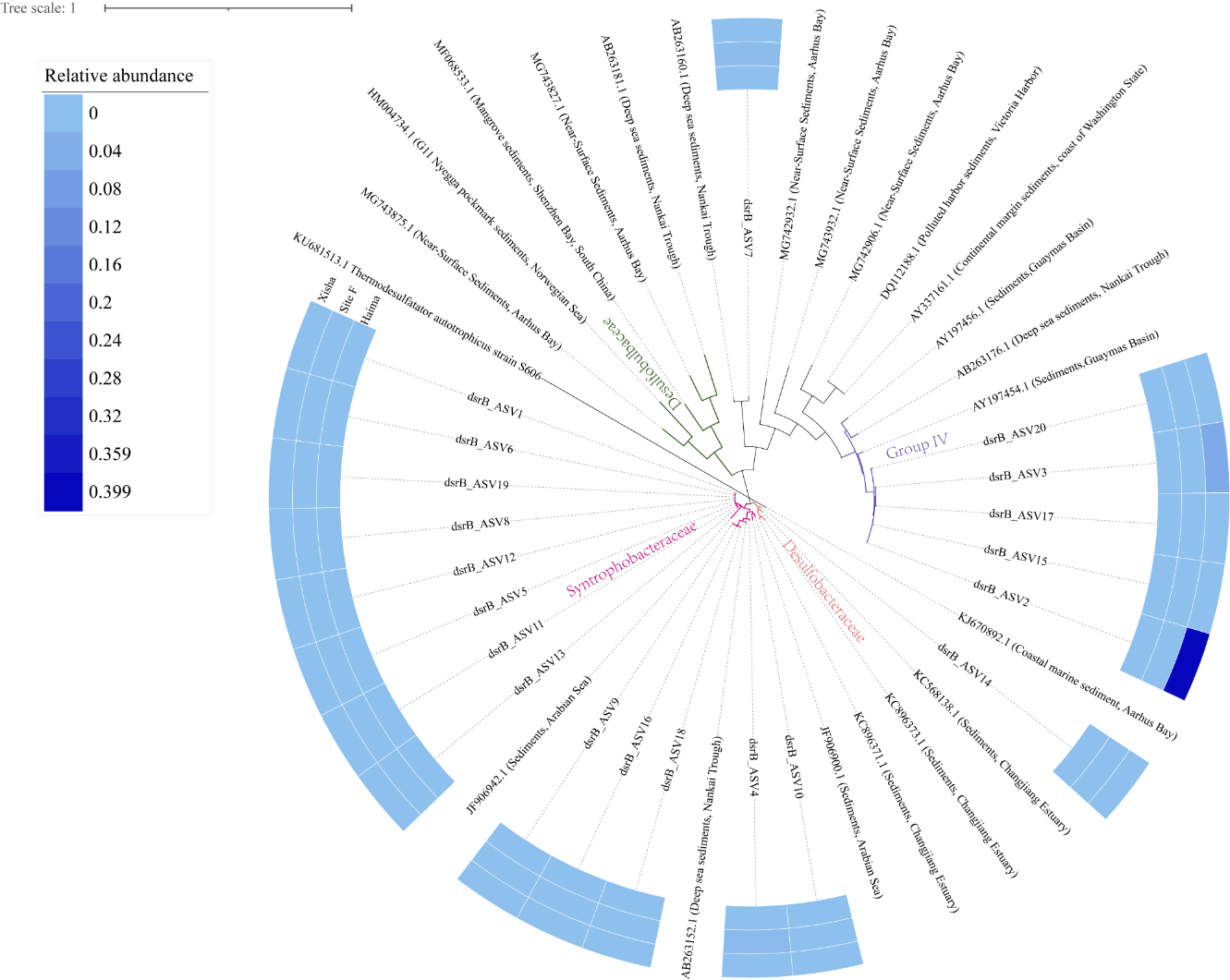


**Figure S4.** Maximum-likelihood phylogenetic trees for *dsr*B gene of top 20 ASVs (accounting for > 97.1% of the total retrieved *dsr*B gene sequences) ^13^CH_4_ + SO_4_^2-^ treatment groups (C). Bootstrap values over 50% based on 1,000 replicates were shown. The abundance of the ASVs in each region was shown in the heatmap referred to the color key.
